# Supplementary material for: Untargeted Metabolomics in Forensic Toxicology: A New Approach for the Detection of Fentanyl Intake in Urine Samples
Source: Molecules. 2021 Aug 18;26(16):4990. doi: 10.3390/molecules26164990 (PMC8398448; doi:10.3390/molecules26164990)
Supplement: Supplementary file 1 [file molecules-26-04990-s001.zip › molecules-1309642-supplementary.pdf]

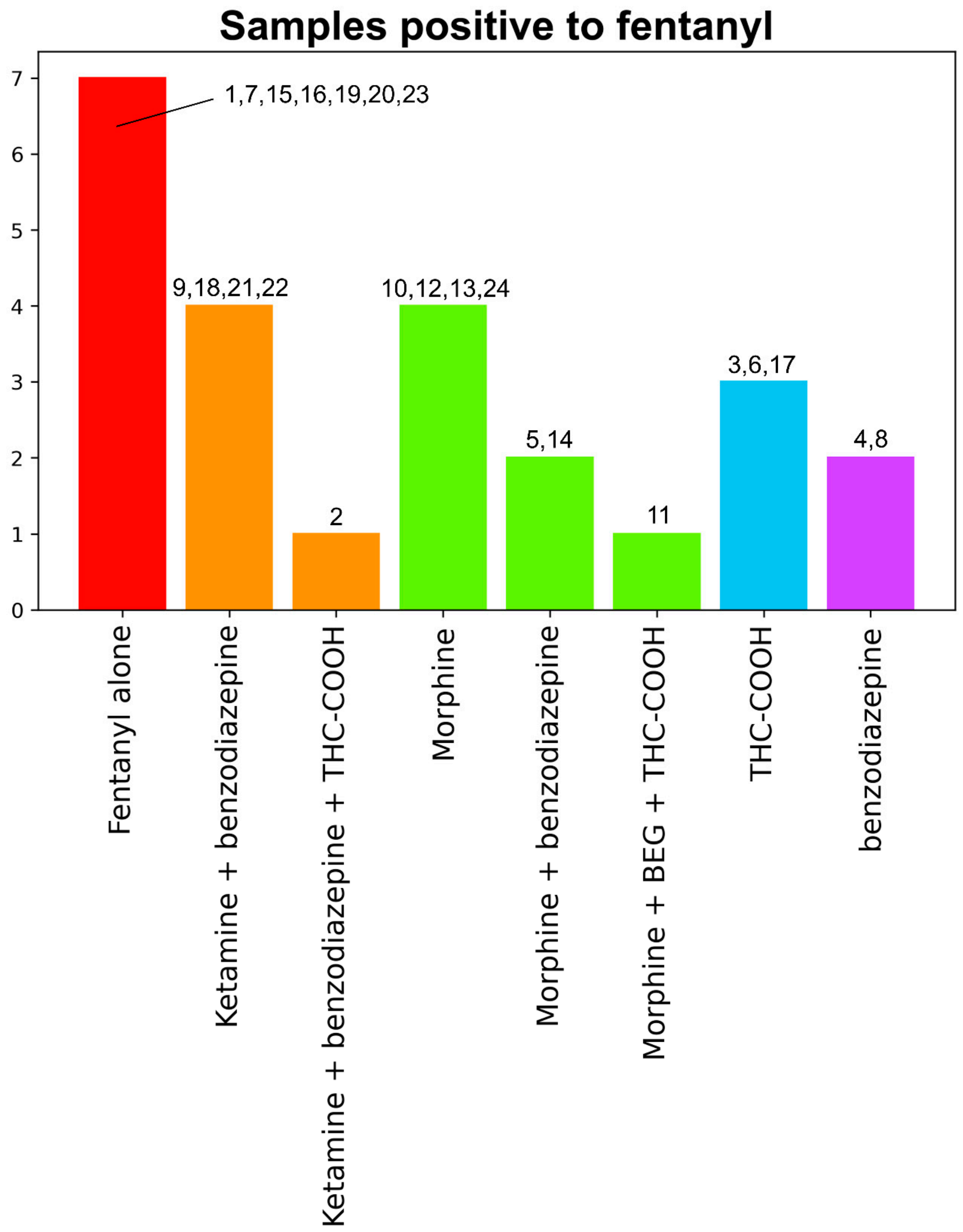

Figure S1. Bar plot reporting the samples positive to fentanyl alone and the ones involved in poly-consumptions. The numbers reported on the bars correspond to the samples' IDs.

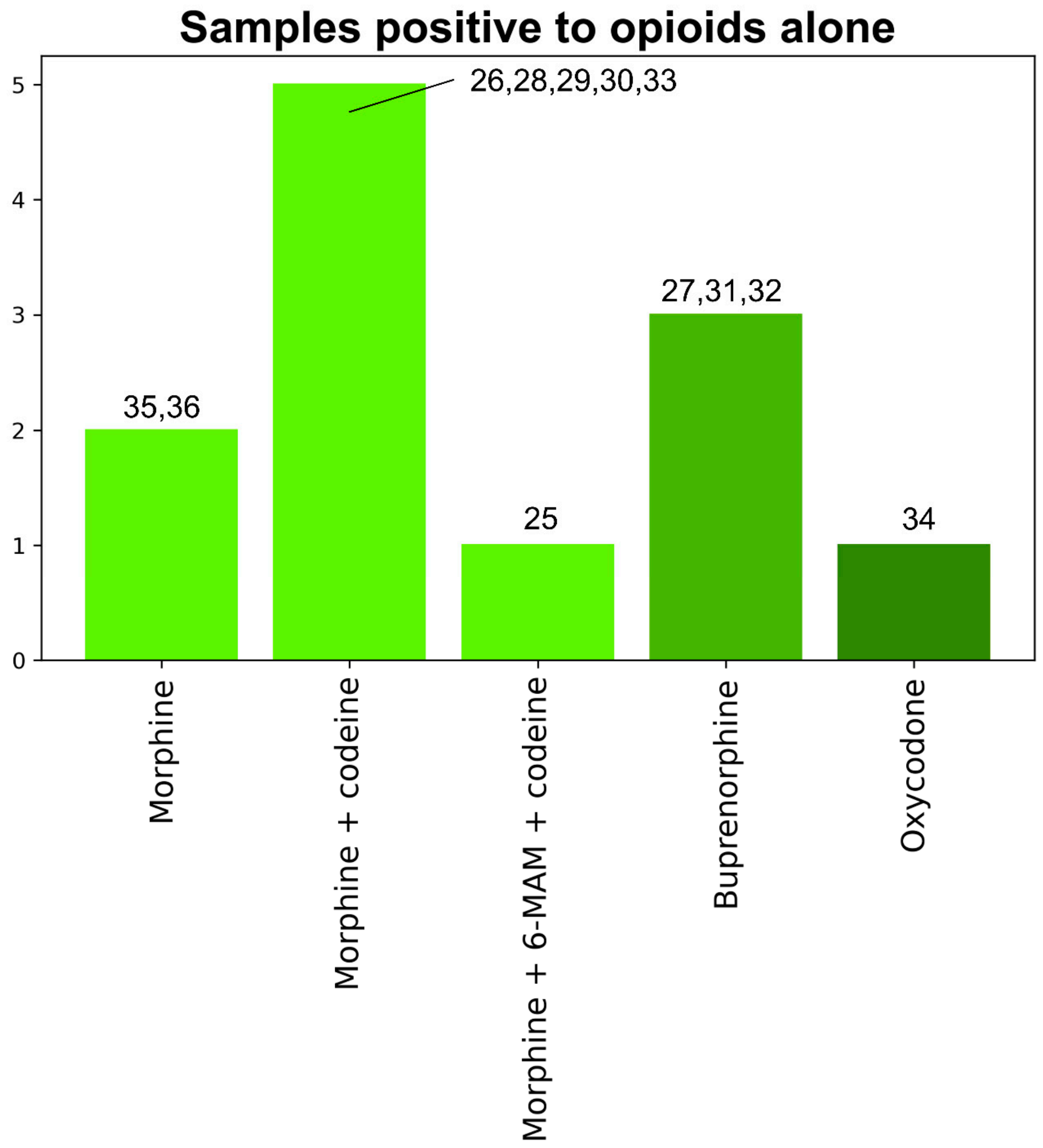

Figure S2. Bar plot reporting the distribution of the samples positive to traditional opioids. The numbers reported on the bars correspond to the samples' IDs.

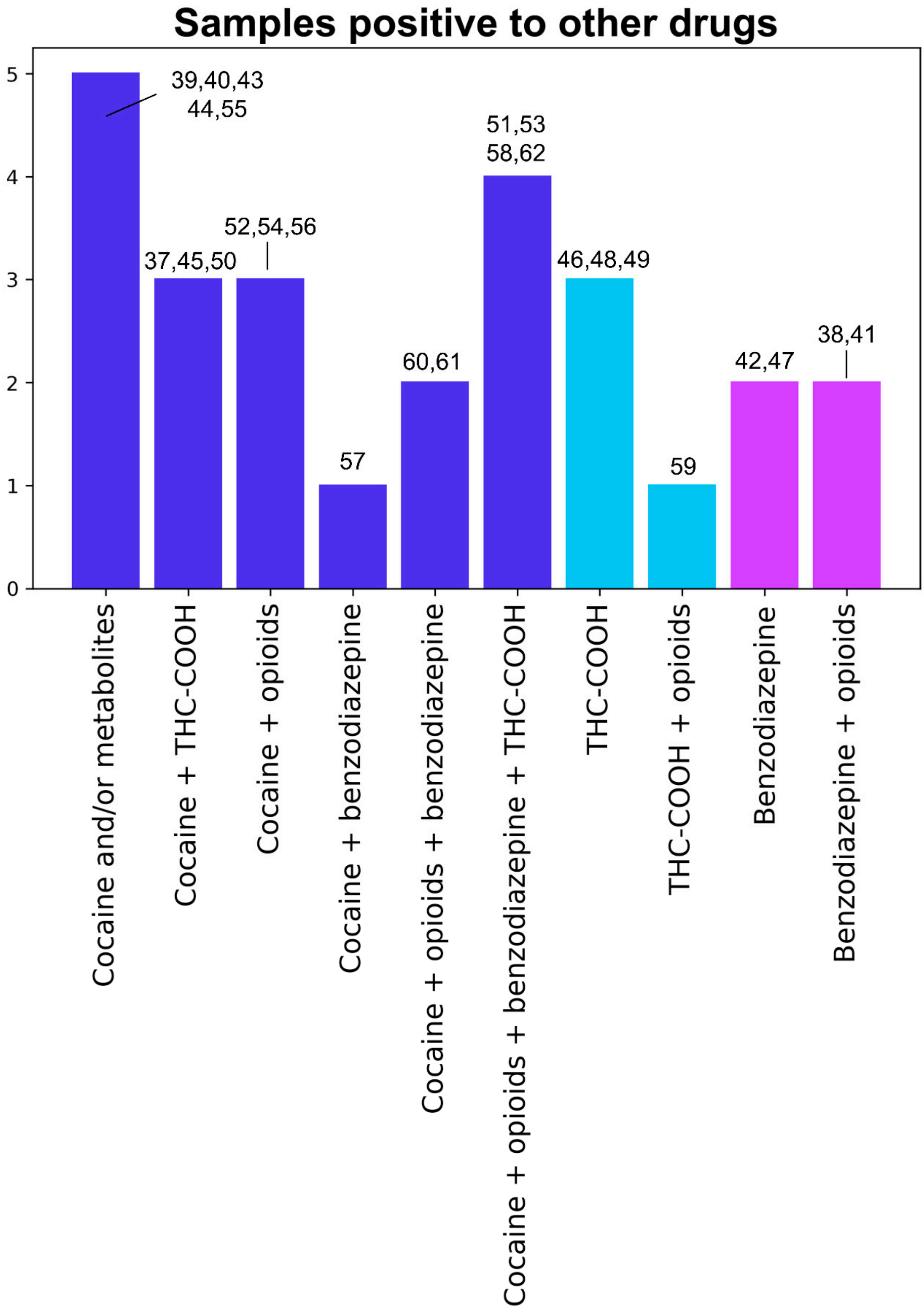

Figure S3. Bar plot reporting the distribution of the samples positive to traditional drugs. The numbers reported on the bars correspond to the samples' IDs.

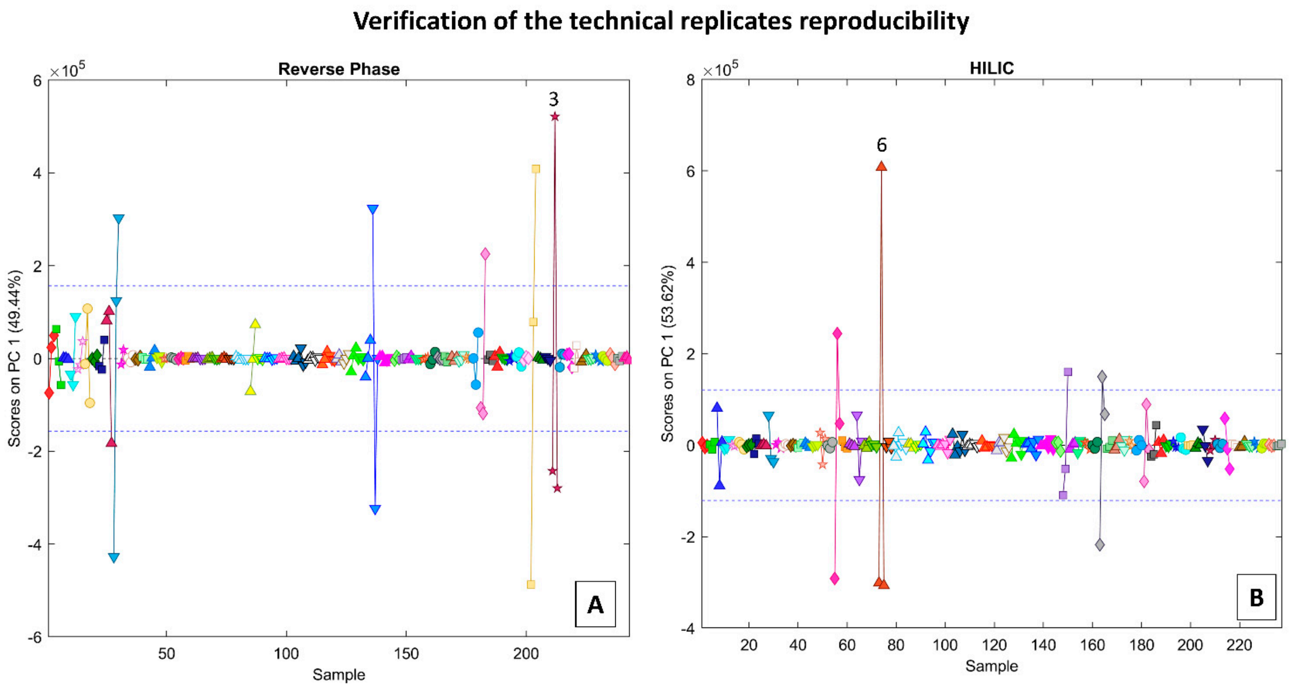

Figure S4. PCA models calculated using the class-centering pre-treatment to investigate the reproducibility of the technical replicates. Each sample is differently colored and the technical replicates are linked by a line. (A) RP experiments and (B) HILIC experiments.

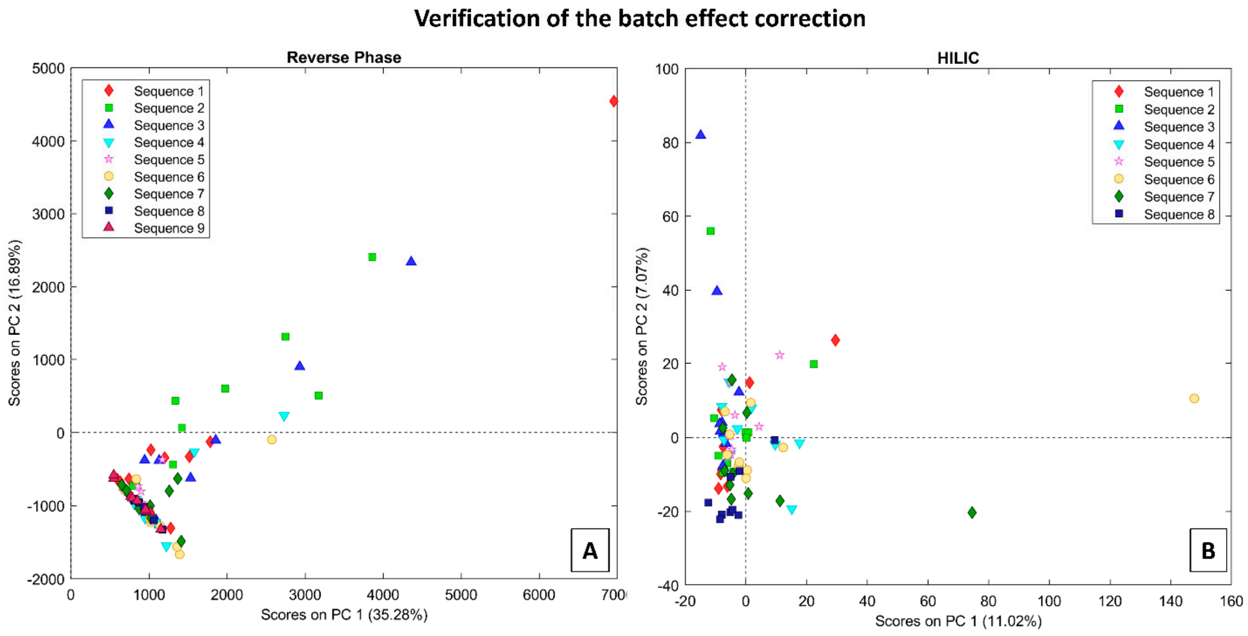

Figure S5. PCA model calculated on the Pareto scaled RP dataset (A) and of the autoscaled HILIC dataset (B). The data-points are coloured on the basis of the analytical sequence.

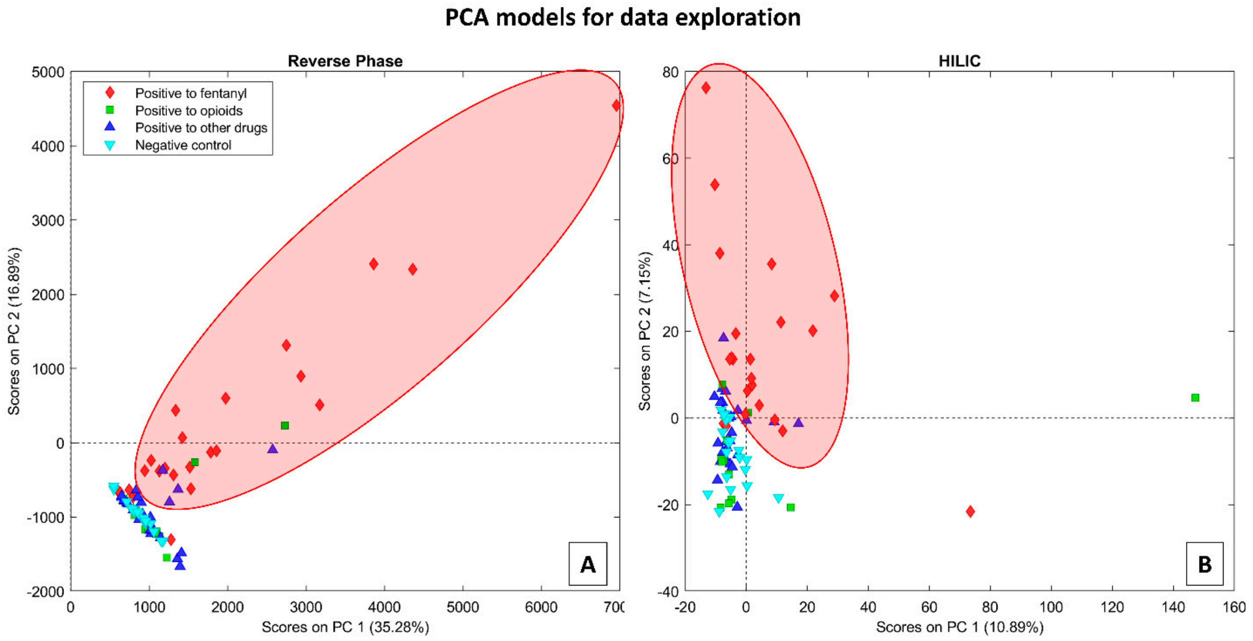

Figure S6. PCA model calculated on the Pareto scaled RP dataset (A) and of the autoscaled HILIC dataset (B). The data-points are coloured on the basis of the four groups identified (see Materials and Methods).

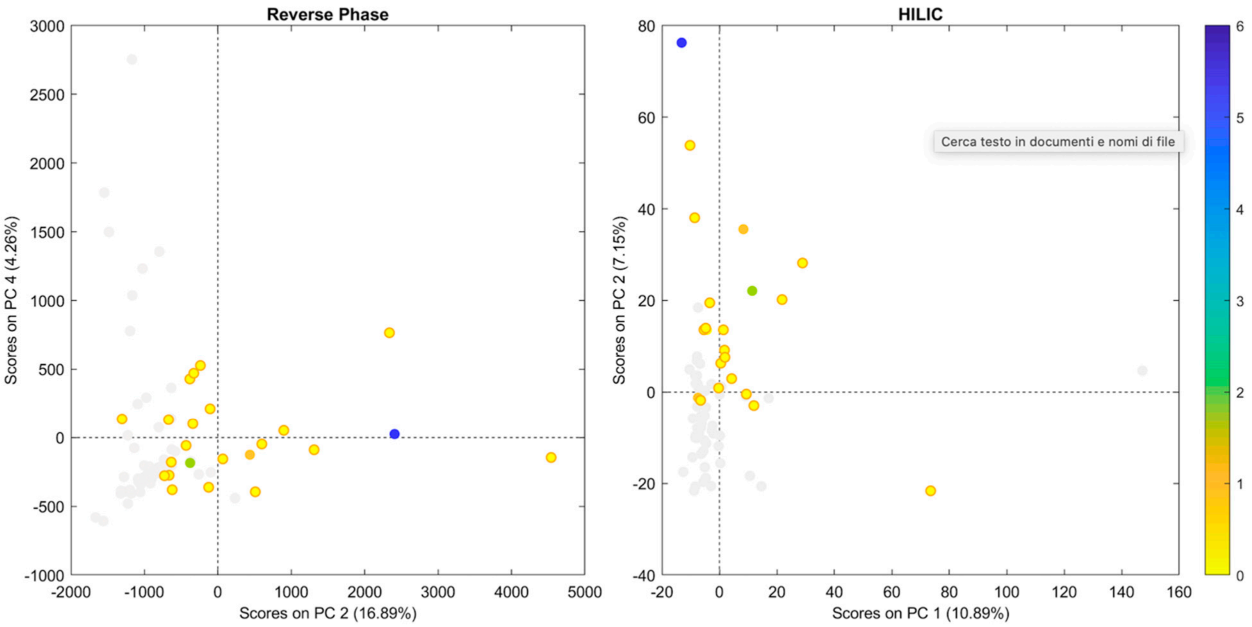

Figure S7. Investigation of a trend related to the fentanyl urinary dosage in the RP (A) and HILIC (B) dataset, respectively.

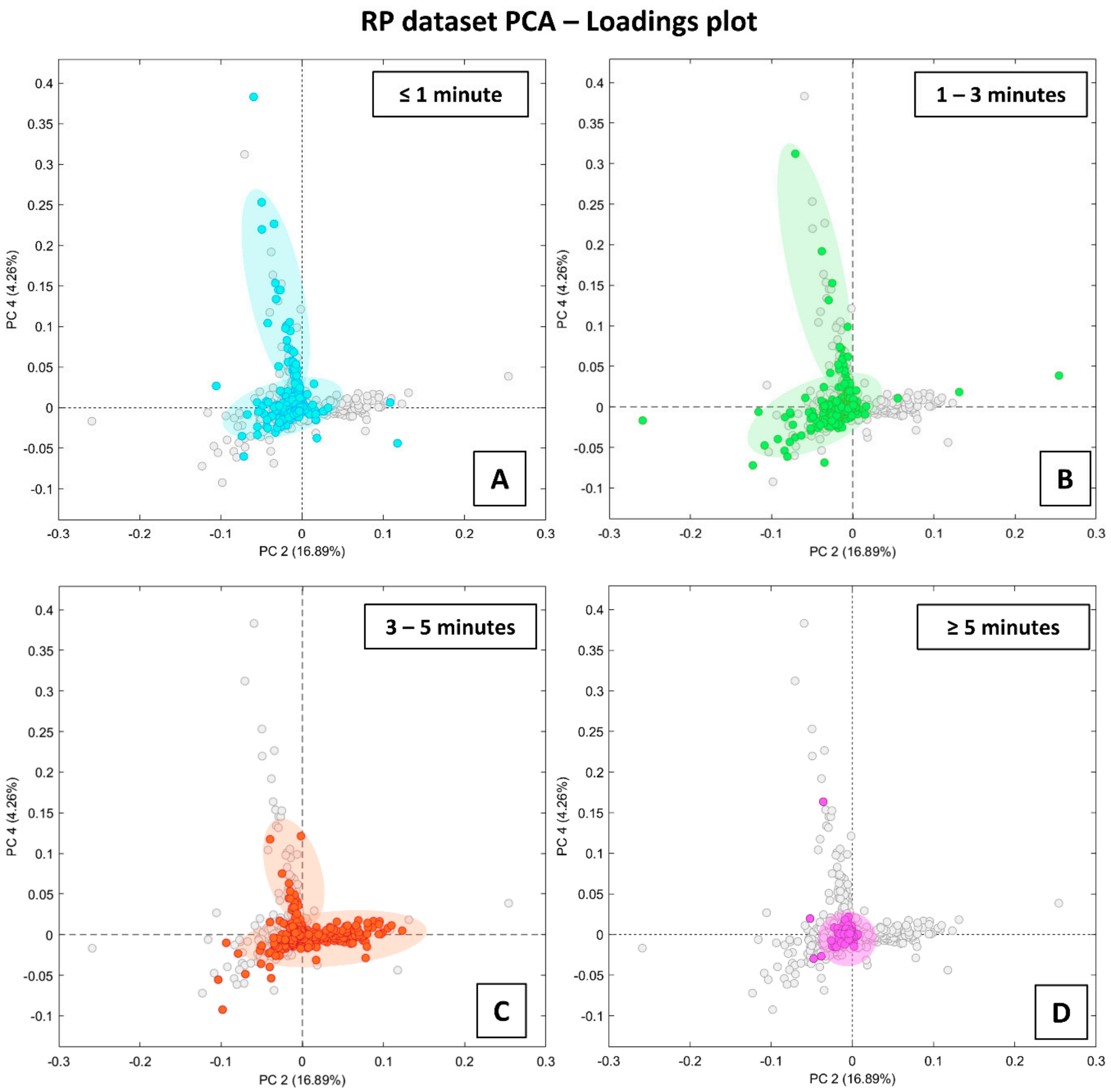

Figure S8. PC2 vs PC4 loadings plot of the PCA model built with the RP dataset. The loadings are coloured on the basis of the retention time: (A) lower than (or equal to) 1 minute, (B) between 1 minute and 3 minutes, (C) between 3 minutes and 5 minutes, and (D) higher than 5 minutes.

Table S1. Fentanyl and norfentanyl dosage. The method's limit of detection is equal to 0.2 ng/mL

#ID	Fentanyl (ng/mL)	Norfentanyl (ng/mL)
1	120	220
2	100	210
3	70	64
4	25	6.0
5	25	68
6	21	59
7	14	6.9
8	14	44
9	14	15
10	12	3.7
11	4.6	2.3
12	4.2	1.9
13	3.0	2.0
14	2.3	1.4
15	2.3	9.7
16	2.1	1.9
17	1.9	20
18	1.0	1.1
19	0.9	3.1
20	0.9	0.5
21	0.9	10
22	0.7	0.5
23	0.4	1.2
24	0.2	<LOD
